# The Tyrosyl-DNA Phosphodiesterase 1β (*Tdp1β*) Gene Discloses an Early Response to Abiotic Stresses

**DOI:** 10.3390/genes8110305

**Published:** 2017-11-03

**Authors:** Maria Elisa Sabatini, Andrea Pagano, Susana Araùjo, Alma Balestrazzi, Anca Macovei

**Affiliations:** 1 Department of Biology and Biotechnology ‘L. Spallanzani’, via Ferrata 9, 27100 Pavia, Italy; maeli_89@hotmail.com (M.E.S.); andrea.pagano01@universitadipavia.it (A.P.); ssaraujo@gmail.com (S.A.); alma.balestrazzi@unipv.it (A.B.); 2 Plant Cell Biotechnology Laboratory, Instituto de Tecnologia Química e Biológica António Xavier, Universidade Nova de Lisboa (ITQB-UNL), Avenida da República, EAN, 2780-157 Oeiras, Portugal

**Keywords:** abiotic stress, *Arabidopsis thaliana*, HIRAN domain, *Medicago truncatula*, tyrosyl-DNA phosphodiesterase

## Abstract

Tyrosyl-DNA phosphodiesterase 1 (Tdp1) is involved in DNA repair pathways as it mends the topoisomerase I—DNA covalent complexes. In plants, a small *Tdp1* gene family, composed by *Tdp1α* and *Tdp1β* genes, was identified, but the roles of these genes in abiotic stress responses are not fully understood. To investigate their specific stress response patterns, the present study made use of bioinformatic and molecular tools to look into the *Tdp1β* gene function, so far described only in the plant kingdom, and compare it with *Tdp1α* gene coding for the canonical, highly conserved α isoform. The expression profiles of *Tdp1α* and *Tdp1β* genes were examined under abiotic stress conditions (cold, heat, high osmolarity, salt, and UV-B) in two model species, *Arabidopsis thaliana* and *Medicago truncatula*. The two isoforms of topoisomerase I (*TOP1α* and *TOP1β*) were also taken into consideration in view of their known roles in DNA metabolism and cell proliferation. Data relative to gene expression in *Arabidopsis* were retrieved from the AtGenExpress microarray dataset, while quantitative Real-Time PCR was carried out to evaluate the stress response in *M. truncatula* cell cultures. These analyses revealed that *Tdp1β* gene expression was enhanced during the first hour of treatment, whereas *Tdp1α* enhanced expression succeeded at subsequent timepoints. In agreement with the gene-specific responses to abiotic stress conditions, the promoter regions of *Tdp1α* and *Tdp1β* genes are well equipped with stress-related *cis*-elements. An in-depth bioinformatic characterization of the HIRAN motif, a distinctive feature of the *Tdp1β* protein, showed its wide distribution in chromatin remodeling and DNA repair proteins. The reported data suggests that *Tdp1β* functions in the early response to abiotic stresses.

## 1. Introduction

Tyrosyl-DNA phosphodiesterase 1 (Tdp1, EC: 3.1.4.-) breaks the covalent 3′-phosphotyrosyl bond between the DNA termini and the catalytic tyrosine residue of DNA topoisomerase I (topo I), removing the highly cytotoxic stabilized topoisomerase I—DNA covalent complexes that impair DNA replication and transcription [1]. Differently from animals, two distinct *Tdp1* isoforms are found in plants, as reported by Macovei et al. [2] in the model legume *Medicago truncatula*. The MtTdp1α amino acid sequence shows similarity with the animal Tdp1 enzyme, while the MtTdp1β protein contains a HIRAN (HIP116 Rad5p N-terminal) domain whose function is still poorly understood [2]. The two isoforms are encoded by *MtTdp1α* and *MtTdp1β* genes, which are upregulated in *M. truncatula* plants in response to heavy metal and osmotic stresses as well as during seed imbibition [2,3,4]. Additional work provided further information on the possible involvement of the *Tdp1β* gene in the plant stress response. Santos et al. [5] showed the upregulation of *Tdp1β* gene in *Medicago sativa* suspension cultures exposed to genotoxic doses of CdSe/ZnS quantum dots, highlighting a strong correlation with DNA damage accumulation/repair kinetics. An RNA-Seq analysis carried out in *MtTdp1α*-depleted *M. truncatula* plants revealed that the *MtTdp1β* gene does not play a redundant function since the *β* isoform was not able to compensate for the lack of *MtTdp1α* gene *in planta* [6]. Even though the *Tdp1β* gene was upregulated in the depleted lines, the overall *Tdp1*(α + β) transcript was reduced, leading to the dwarf phenotype resulted from the *Tdp1α* depletion [6].

The specific role of the *Tdp1β* gene in plants remains unclear. Here, we propose to investigate the expression profiles of the *Tdp1* gene family in response to multiple abiotic stress agents in two model plants, *Arabidopsis thaliana* (thale cress) and *Medicago truncatula* (barrel medic). The two different model species, belonging to *Brassicaceae* and *Leguminosae* families, respectively, were used to evaluate the conservative response among species. The promoter regions of *Tdp1α* and *Tdp1β* genes were also examined with bioinformatic tools. Additionally, the presence and distribution of the HIRAN domain in the plant kingdom were investigated.

## 2. Materials and Methods

### 2.1. Plant Material and Treatments

Suspension cultures of *M. truncatula* cv. Jemalong, M9-10a genotype were propagated in liquid Murashige-Skoog (MS) medium [7] containing 3% (*w*/*v*) sucrose, 0.5 mg L^−1^ 2,4-dichlorophenoxyacetic acid (2,4-D, Micropoli, Milan, Italy), and 0.5 mg L^−1^ kinetin (Micropoli), at pH 5.7. The suspension cultures were sub-cultured every eight days and maintained at 23 °C, in agitation (80 rpm) under dark conditions. Highly proliferating four-day-old suspension cultures were used for further treatments. To perform the stress treatments, 10 mL suspension culture was transferred to 50 mL Falcon tubes, maintained in agitation. The following treatments were imposed to the cell suspensions: salt (NaCl, 250 mM), high osmolarity (PEG6000, 150 g L^−1^), cold (4 °C), heat (42 °C), and UV-B (15 min exposure to 280–315 nm). For the NaCl and PEG treatments, the solutions were added to the liquid MS medium. For the cold and heat treatments, the tubes were incubated at the indicated temperatures. Exposure to UV-B was performed in opened Petri dishes, containing the same volume of suspension, which was circularly moved every 2 min during the time of exposure. For all treatments, samples were collected at 0 h, 0.5 h (30 min), 1 h, 3 h, and 6 h following exposure.

### 2.2. Detection of Cell Death

Loss of plasma membrane integrity was assessed through Evans Blue staining, as previously described [8]. Proliferating *M. truncatula* cell cultures subjected to the abovementioned treatments for 6 h were used for this analysis. Briefly, Evans Blue (Sigma–Aldrich, Milan, Italy) stock solution (10 mg/mL) was added to cell suspension samples (1 mL) to a final concentration of 0.025% (*v*/*v*). After 10 min of incubation at room temperature, the culture was extensively washed with distilled water to remove excess and unbound dye. Subsequently, Evans Blue bound to dead cells was extracted using 50% (*v*/*v*) methanol with 1% (*w*/*v*) SDS at 60 °C for 30 min and the absorbance was read at 600 nm using a Jasco 7800 UV/Vis Spectrophotometer (JASCO, Easton, MD, USA). Non-treated samples and cells subjected to heat-shock (HS, 65 °C for 10 min) were used and positive and negative controls, respectively. Images were recorded using an Olympus SZX9 Stereomicroscope (Olympus Italia S.R.L., Milan, Italy). For each treatment combination, three independent and two biological replicas were used. Results are presented as % of cell death, where absorbance of heat-shock (HS) treated cells is considered as 100%. The results were statistically evaluated by *t*-test (*, *p* < 0.05), with a non-treated control taken as a reference.

### 2.3. Gene Expression Analysis

The *AtTdp1α* (GB# FJ858738) and *AtTdp1β* (GB# BT006446.1) gene expression profiles were retrieved from the AtGenExpress microarray abiotic stress dataset [9]. This data provides the global transcriptome of *Arabidopsis* shoots challenged with cold (TAIR accession ME00325), heat (TAIR accession ME00338), high osmolarity (TAIR accession ME00327), salt (TAIR accession ME00328), and UV-B light (TAIR accession ME00329).

To evaluate the expression of *MtTdp1α* (GB# AC122166), *MtTdp1β* (GB# AC141864.7), and *MtTOP1α* (GB# CA919655), *MtTOP1β* (GB# CX526330), RNA extraction, cDNA synthesis, and qRT-PCR analysis were carried out. The *M. truncatula ELF1α* (GB# EST317575) was used as a reference gene [6,10,11]. Total RNA was isolated as previously described [12]. One microgram of RNA was reverse-transcribed using the RevertAid First Strand cDNA Synthesis Kit (Thermo Fisher, Monza, Italy). qRT-PCR was carried out using the Maxima SYBR Green qPCR Master Mix (Thermo Fisher, Monza, Italy). The gene-specific oligonucleotide primers used in this study were: FW 5′-ACGAGTTGGGAGTGCTCTTT-3′, REV: 5′-GGGATTTATCCTTCGATTGTTT-3′ for *MtTdp1α*, FW: 5′-GGTTGGTTTGAGCCATCTTT -3′, REV: 5′- GCAGGCACATTGTGATTTCT-3′ for *MtTdp1β*; FW: 5′-AAACTGACATCGGGAGGAAC-3′, REV: 5′-TTCTGCTTCACCCAGTCATC-3′ for *MtTOP1α*; FW: 5′-ATACACGTGGGCTATTGTCG-3′, and REV: 5′-TCACTTGGATGAATGCGTT-3′ for *MtTOP1β*; and FW: 5′-GACAAGCGTGTGATCGAGAGATT-3′, REV: 5′-TTTCACGCTCAGCCTTAAGCT-3′ for *MtELF1α*. For each oligonucleotide set, a no-template control was used. Amplification conditions were as follows: initial denaturation step at 95 °C for 30 s, and subsequently 95 °C for 5 s, 60 °C for 30 s, and 72 °C for 30 s (40 cycles). C_t_ values and qRT-PCR efficiency values, obtained by the Rotor-Gene 6000 Series Software 1.7 (Corbett Robotics, Brisbane, Australia), were analyzed and statistically validated using the REST2009 Software V2.0.13 (Qiagen GmbH, Hilden, Germany).

For both *Arabidopsis* and *Medicago* collected data, the selected timepoints per treatment were 0 h, 0.5 h (30 min), 1 h, 3 h, and 6 h after treatment. The data is presented as fold change to control (untreated samples), with each timepoint being normalized to its corresponding control. The data not normalized to the untreated control is provided in Supplementary Tables S1 and S2, respectively. MeV (Multiple Experiment Viewer) software (http://mev.tm4.org) was used to represent the data.

### 2.4. Promoter Analysis

The PlantCARE database [13] was used for the in silico analysis of promoter sequences. Sequences corresponding to 1500 bp upstream of the start codon were retrieved from NCBI for each gene. The percentage of stress-related *cis*-elements was calculated based on the total number of elements identified for each promoter region. A Venn diagram (http://bioinformatics.psb.ugent.be/webtools/Venn/) analysis was used to assess the number of common vs. specific elements for each promoter sequence.

### 2.5. Phylogenetic Tree Analysis

To investigate the occurrence of HIRAN domain in the plant kingdom, the *Tdp1β* amino acid sequences were compared using the ExPaSy SIB BLAST Network Service (http://www.expasy.ch/tools/blast//) and the EMBL-EBI Clustal W2 Multiple Sequence Alignment (http://www.ebi.ac.uk/tools/msa/clustalw2) tools. The phylogenetic tree was built using the Plaza 2.5 tool (bioinformatics.psb.ugent.be/plaza/) [14].

## 3. Results

### 3.1. Effect of Treatments on Cell Viability

**Figure 1:**
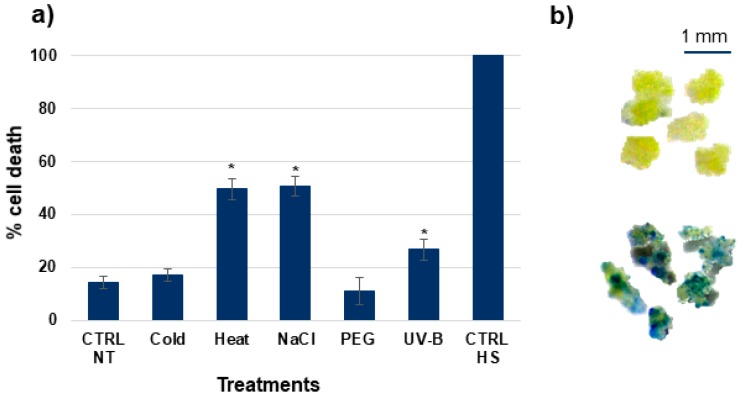
Evaluation of cellular damage and deterioration of membrane permeability by Evans Blue staining. (**a**) Percentage of cell death in *Medicago truncatula* cultures treated with cold, heat, NaCl, PEG, and UV-B for 6 h; CTRL NT, non-treated control; CTRL HS, heat-shock treated negative control; (**b**) Morphology of *M. truncatula* cell aggregates (upper image) and cells stained with Evans Blue (lower image).

*M. truncatula* cell cultures treated with cold, heat, PEG, NaCl, UV-B, and respective positive (CTRL NT) and negative (CTRL HS) controls, were subjected to Evans Blue staining to evaluate cellular mortality (Figure 1). Measurements were taken after 6 h of treatment, because this is indicated as the minimum threshold required for the induction of programmed cell death [15]. The treatments that most affected cell viability were heat, NaCl, and UV-B, while PEG and cold treatments did not show significant changes compared to the non-treated control (CTRL NT) (Figure 1a). *M. truncatula* M9-10a suspension cultures are composed mainly of microcalli (Figure 1b), in accordance with a previous description defining them as highly embryogenic cell aggregates of small spherical cells [16].

### 3.2. The Expression Profiles of Both Tdp1β and Tdp1α Genes Change during Abiotic Stress Treatments

**Figure 2:**
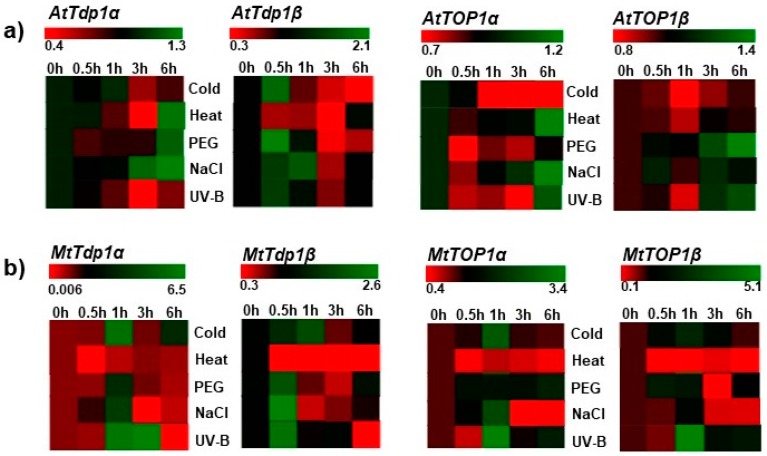
Expression profiles of the *Tdp1* and *TOP1* gene families in *Arabidopsis thaliana* (**a**) and *M. truncatula* (**b**). The heatmaps were generated using MeV (Multiple Experiment Viewer) online software (http://mev.tm4.org). Data are presented as fold change to the untreated control for each timepoint.

The expression levels of *Tdp1α* and *Tdp1β* genes were evaluated in response to various abiotic stress conditions in two different model plants, *A. thaliana* and *M. truncatula* (Figure 2). The analysis of *Arabidopsis* gene expression in shoots shows that the strongest expression (green colour) of *AtTdp1α* corresponds to 6 h following exposure to heat and high osmolarity, and 3–6 h after treatment with salt (Figure 2a, *AtTdp1α*; Supplementary Table S1). Conversely, the lowest expression (red colour) is observed at 3 h following exposure to heat and UV-B. When considering the expression profiles of *AtTdp1β*, the strongest expression is evident mostly within the first hours of treatment (0.5 h high osmolarity, salt, cold, UV-B, and 1 h salt stress). The lowest gene expression is associated with exposure to heat stress (at 0.5 h, 1 h, and 3 h), as well as at later timepoints after cold (3 h and 6 h) and UV-B (3 h) treatments (Figure 2a, *AtTdp1β*; Supplementary Table S1).

To assess whether the early response to abiotic stress observed in *Arabidopsis* is a conserved feature of the plant *Tdp1β* gene, the expression profiles of the *Tdp1* gene family were analyzed in a different system, namely actively proliferating *M. truncatula* in vitro cell cultures. Also in this case, the *MtTdp1β* gene expression is strongest soon after the beginning of the treatment (0.5 and 1 h) with salt, high osmolarity agent, and UV-B, while heat stress resulted in the lowest expression (Figure 2b, *MtTdp1β*; Supplementary Table S2). The *MtTdp1α* gene expression was induced after 1 h (cold, PEG, NaCl, UV-B), 3 h (UV-B) and 6 h (cold) of treatment (Figure 2b, *MtTdp1α*; Supplementary Table S2).

The early (30 min to 1 h) expression profiles of the *Tdp1β* gene are thus maintained in both *A. thaliana* and *M. truncatula* model systems. Differences in the expression patterns in response to time and treatments could be due to the use of different methods to quantify gene expression (microarray in *Arabidopsis* and qRT-PCR in barrel medic) and different plant material (shoots vs. cell suspensions).

To assure that the chosen treatments affect DNA metabolism and cell proliferation, the expression of topoisomerase I α and β isoforms was also evaluated. In the *Arabidopsis* system, *AtTOP1α* is mostly downregulated, while upregulation is evident only after 6 h of treatment with heat, NaCl, and UV-B (Figure 2a, *AtTOP1α*; Supplementary Table S1). *AtTOP1β* is highly expressed both at 3 and 6 h after treatment with PEG, NaCl, and UV-B (Figure 2a, *AtTOP1β*; Supplementary Table S1). When considering the cell suspension system, both *MtTOP1α* and *MtTOP1β* were highly expressed after 1 h of treatment, and mostly downregulated following the 1 h time point (Figure 2b, Supplementary Table S2). In is important to note that the *MtTdp1* and *MtTOP1* genes followed similar patterns of expression in *M. truncatula* cell cultures (e.g., upregulation during the first hours of treatment vs. downregulation at later timepoints). Another point is that all genes were downregulated during treatment with heat, which also resulted in high cell mortality rates, indicating that high temperature is the most repressive affliction in our cell culture system.

### 3.3. In Silico Analysis of Tdp1β and Tdp1α Promoter Sequences Reveals Abundant Stress-Related cis-Elements

**Figure 3:**
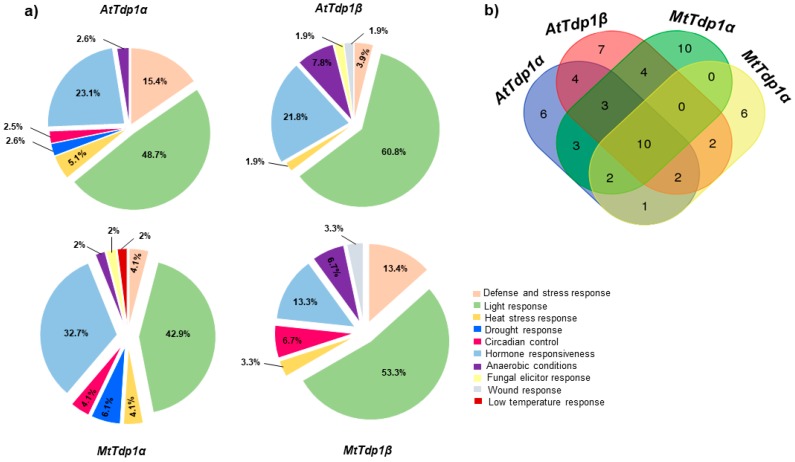
Distribution of *cis*-elements in the promoter region (1500 bp upstream the start codon) of *AtTdp1α, AtTdp1β, MtTdp1α*, and *MtTdp1β* genes. (**a**) Percentage of stress-related *cis*-elements; (**b**) Venn diagram presenting the number of common and different elements per promoter region.

The *cis*-elements found in the four promoter regions (1500 bp upstream from the start codon) of *AtTdp1α*, *AtTdp1β*, *MtTdp1α*, and *MtTdp1β* genes are quite diverse. Nonetheless, all four promoters are well equipped with stress-related *cis*-elements (Figure 3). Among these, the light-responsive and hormone-responsive elements are the most abundant. Another well-represented class belongs to the defense and stress response, accounting for 15.4% and 13.4% of the overall *cis*-elements in *AtTdp1α* and *MtTdp1β* (Figure 3a). To assess the number of common and different *cis*-elements per promoter, a Venn diagram was constructed. The analysis showed that 10 *cis*-elements are common to all four promoters (Figure 3b). These include *cis*-elements involved in anaerobic induction (ARE), light response (Box 4, Box I, G boxes), heat stress (HSE), salicylic acid response (TCA-element), endosperm-specific expression (Snk1_motif), as well as *cis*-regulatory elements which act as enhancer (CAAT-box) and transcription factors binding sites (TATA-box) (Supplementary Table S3). When considering the elements specific to each promoter region, the *MtTdp1α* has the highest number of specific elements (10) when compared with the other promoter regions. These include elements involved in auxin response (TGA-element, AuxRR-core), meristem-specific expression (CAT-box), low temperature (LTR) and light response (3-AF1 binding site, GATA-motif, GTGGC-motif, I-box), the binding site of AT-rich DNA binding protein (ATBP-1) (AT-rich element), and the *cis*-acting regulatory element, the OBP-1 site. In the *AtTdp1α* promoter region, the six specific elements are involved in abscisic acid (ABA) and viviparous1 (VP1) responsiveness (CE3), lignin biosynthesis (AC I, AC II), and light response (GA-motif, Gap-box, Box II). The detected elements specific only to *AtTdp1β* include *cis*-elements involved in gibberellin response (GARE-motif), differentiation of the palisade mesophyll cells (HD-Zip I), control of leaf morphology (HD-Zip 2), and light response (4cl-CMA2b, CATT-motif, MRE). Lastly, the specific *cis*-elements encountered in the *MtTdp1β* promoter regions are involved in gibberellin (P-box) and light response (chs-CMA2a, GAG-motif, ATCT-motif, AT1-motif).

Overall, the promoter region of the four genes investigated are both similar and diverse, considering the type and number of *cis*-elements present, respectively.

### 3.4. The HIRAN Domain is Ubiquitously Distributed in Plants

**Figure 4:**
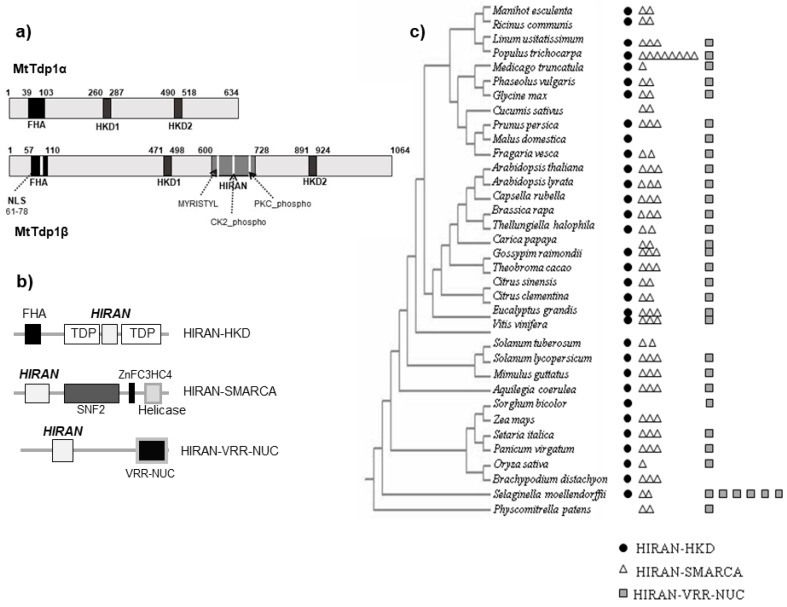
In silico analysis of the HIRAN (HIP116 Rad5p N-terminal) domain in plants. (**a**) Schematic representation of *MtTdp1α* and *MtTdp1β* proteins; NLS, nuclear localization signal; (**b**) Different domain organizations of the HIRAN motif; (**c**) Phylogenetic distribution of the HIRAN-HKD, HIRAN-SMARCA, and HIRAN-VRR-NUC domain organizations in the plant kingdom.

A detailed bioinformatic investigation of the HIRAN domain was performed to gain insights into its distribution in plants (Figure 4). The schematic representation of *MtTdp1α* and *MtTdpβ* protein sequence evidences the presence of the HKD (HxKx(4)-D-x(6)-G-S-x-N) catalytic sites in both proteins, while the HIRAN domain is found only in the β isoform (Figure 4a); in addition, a nuclear localization signal (NLS) is also present only in the β isoform within the ForkHead-Associated (FHA) domain. Three different domain organizations of the HIRAN motif (HIRAN-HKD, HIRAN-SMARCA, HIRAN-VRR-NUC) are distributed throughout the plant kingdom (Figure 4b). In the HIRAN-HKD structure, present in the plant *Tdp1β* protein, the HIRAN motif is flanked by two interacting HKD motifs, producing an active site for the phosphodiesterase activity [1]. The HIRAN-HKD organization includes also an FHA motif, present in several eukaryotic nuclear proteins, which mediates phosphorylation-dependent protein-protein interactions [17]. The FHA domain recognizes phosphopeptides arising from hyperphosphorylation mediated by cell-cycle checkpoint kinases in response to DNA damage [17]. The HIRAN-SMARCA domain organization is typical for the A-SMARC (SWI/SNF-Related, Matrix-associated, Actin-dependent Regulator Chromatin) group of proteins, represented by DNA-dependent ATPases able to modify histone-DNA interactions and modulate chromatin organization [18]. It also includes other domains, e.g., the SNF2 involved in transcription regulation, recombination, and chromatin remodeling [19], and the C3HC4 type zinc-finger motif with functions in the ubiquitination pathway [20]. Additionally, the HIRAN-SMARCA domain organization is found in the Helicase C-terminal motif, typical for the SF1 and SF2 superfamilies [21]. The HIRAN-VRR-NUC domain, poorly characterized in plants, is found in human cells within the highly conserved protein KIAA1018/FAN1 (Fanconi anemia-Associated Nuclease 1), a DNA repair nuclease recruited to damaged DNA [22] which localizes to stalled replication forks coordinating S-phase arrest and DNA repair [23]. In plants, this domain is present in the KIAA1018-like protein in association with an N-terminal Rad18 zinc finger region and C-terminal tetratricopeptide repeats (TPRs) [24].

The phylogenetic analysis shows that the HIRAN-HKD domain organization is detected in Dicots, such as the model plant *A. thaliana*, the legumes *M. truncatula* and *Glycine max*, and some tropical species (Figure 4c). The HIRAN-HKD domain organization is also present in monocots but absent in the moss *Physcomitrella patens*. Both the HIRAN-SMARCA and HIRAN-VRR-NUC domain organizations are widely distributed in the plant kingdom. Indeed, the most widespread domain structure involving the HIRAN motif in eukaryotes is found in the N-terminus of the SWI2/SNF2 ATPases required for the activation of cell-cycle checkpoints [25].

## 4. Discussion

Plant *Tdp* genes are far less studied when compared with their human counterparts. Moreover, the presence of multiple *Tdp1* genes in plants [2] further expands the need to design dedicated studies to investigate their implication in plant development and stress response. In human cells, *Tdp1* is strongly linked with complex functions in DNA repair [26], whereas mutations in its catalytic sites are associated with serious diseases (e.g., spinocerebellar ataxia) [27]. Studies conducted so far in plants have proven the involvement of the *Tdp1α* gene in DNA damage repair and stress response [2,6,28]. Moreover, the lack of the canonical *Tdp1* isoform (*Tdp1α*) was associated with phenotypic defects (e.g., dwarfism) in both *Arabidopsis* [28] and *M. truncatula* [6]. On the other side, the *Tdp1β* gene is far less characterized. In *M. truncatula*, it was shown to be ubiquitously expressed in all plant tissues and developmental stages [2,3]. As the *MtTdp1β* gene was not able to compensate for the deficiency of *MtTdp1α* gene in *M. truncatula* transgenics [6], we hypothesize that the two genes might not have an overlapping function, although both of them play certain roles in response to abiotic stresses [2]. To further assess this hypothesis, here we investigated the expression profiles of the *Tdp1β* gene, alongside the canonical *Tdp1α* gene, in two model species, *A. thaliana* and *M. truncatula*. Several abiotic stress conditions (cold, heat, salinity, osmotic stress, and UV-B) were imposed. Our analysis showed that while *Tdp1β* is strongly expressed at the earliest timepoints (0.5–1 h) following exposure to stress in both Arabidopsis and *M. truncatula* (Figure 2), the expression of the *Tdp1α* gene is more variegated between the two species. The early stress response pattern of *Tdp1β* gene is conserved between the two species in spite of the different methods used to quantify gene expression (microarray in *Arabidopsis* and qRT-PCR in *M. truncatula*) and different plant material (shoots vs. cell suspensions generated from aerial parts). The use of microarrays to evaluate gene expression levels is quite widespread, while this often requires validation by qRT-PCR. Nonetheless, most studies using both methods agree on a high level of equivalency among them [29,30,31]. When considering the different type of material used, a recent study showed the occurrence of similar mitotic indexes and gene expression profiles in *M. truncatula* leaves grown in a greenhouse and calli-cultured in vitro [32]. Likewise, our work using *M. truncatula* overexpressing the *MtTdp2α* gene revealed a high degree of similarity in the expression pattern of several DNA repair genes in both plants grown in vitro and cell suspension cultures [10,33]. Hence, the differences encountered in the expression levels of *Tdp1α* and *Tdp1β* among *Arabidopsis* and *M. truncatula* are most likely species-specific.

The percentage of cellular mortality and expression of *TOP1* isoforms were assessed to gain more insight into cell behaviour during treatments. As DNA topoisomerase I is involved in solving the conformational changes in DNA topology, it plays essential roles in several cellular processes (e.g., replication, transcription, recombination) [34] as well as in the response to stress agents [35,36,37]. It can also act as a damage sensor and cofactor in DNA repair pathways [38,39]. Moreover, the topo I—DNA covalent complexes represent the substrate for the activity of *Tdp1* [26,40]. Due to their central role in DNA metabolism, decreased activity of the *TOP1* gene negatively influences cell culture growth and vitality [15]. The results presented here corroborate with this finding well, as enhanced cellular mortality (Figure 1) corresponded to a decrease in *MtTOP1α* and *MtTOP1β* gene expression (Figure 2) at 6 h following treatments. Downregulation of *TOP1* could be associated with a temporary block of cell-cycle progression to allow more time for DNA repair. In *M. truncatula* cell cultures, higher expression of *TOP1* genes after 1 h of treatment was observed in parallel with higher expression of *Tdp1* genes, suggesting for an early and coordinated activation of these genes under stress conditions. It is reasonable to hypothesize that these may be acting as damage sensors rather than repair activities, as further exposure to stress (e.g., subsequent timepoints) resulted in decreased gene expression.

The activation or suppression of gene expression is tightly regulated at the transcriptional level through the activity of gene promoters and related *cis*-acting elements. As a consequence, the regulation of gene transcription revolves around the type, number, position, and combination of regulatory elements present inside and around the promoter [41]. As we detected different expression patterns in *Tdp1α* and *Tdp1β* genes in response to stress, we investigated their promoter regions in the two model species. In agreement with the gene-specific responses to abiotic stress conditions, the promoter regions of all four genes are adequately equipped with stress-related *cis*-elements (Figure 3). Common as well as divergent *cis*-elements are encountered in the promoter regions of the four genes. Focusing on the different types of *cis*-elements, the hormone-responsive elements are worth specifying because abiotic stress responses are strongly correlated with hormonal signaling [42]. Our analysis showed that ABA-responsive elements are present only in *AtTdp1α*, and auxin-responsive elements are present only in *MtTdp1α*, while gibberellin-responsive elements are common to both *AtTdp1β* and *MtTdp1β* promoter sequences. On the other hand, salicylic acid-responsive *cis*-elements are encountered in all four promoter regions. It is well established that phytohormones and their corresponding cross-talk pathways play pivotal roles during plant development and stress response [43]. Another point to be raised is the presence of *cis*-elements with roles in lignin biosynthesis in the *AtTdp1α* promoter region. This is important because lignin, as the main structural component of the cell wall, is involved in the overall plant stress management [44]. Moreover, we showed that in *M. truncatula*, *MtTdp1α*-depleted plants have a reduced permeability of the cell wall and ticker cuticula than their wild-type counterparts, and this was associated with altered expression of defense genes and high susceptibility to stress [45]. A recent study showed the presence of common metabolites in the formation of lignin and cuticular biopolymers in mosses, suggesting that the pre-lignin pathway may be crucial for the formation of cuticular elements [46]. However, despite the fact that the *cis*-element promoter analysis represents an informative tool to figure out differences in gene expression profiles, future promoter validation studies are required to corroborate the in silico investigation.

To further unfold the reasons of *Tdp1β* early stress response, we investigated the presence and distribution of the HIRAN domain (conserved in many DNA processing proteins) in plants. This analysis showed that the HIRAN-HKD domain, specific to the Tdp1β protein, along with the HIRAN-SMARCA and HIRAN-VRR-NUC conformations, are ubiquitously found in plants (Figure 4). The structure of the HIRAN domain was recently reported to be composed of six β-strands and two α-helices, forming an OB (oligonucleotide/oligosaccharide binding)-fold structure commonly found in single strand DNA (ssDNA) binding proteins [47], while the DNA binding site of the free domain displayed high degrees of conformational heterogeneity [48]. It has been hypothesized that the HIRAN motif might act in the recruitment of repair/remodeling enzymes to specific DNA sites, playing a role in cell cycle-checkpoints arising from stalled replication forks and post-replication damage [49]. In human cells, the filling-in of gaps in damaged DNA during replication, carried out by the HLTF (helicase-like transcription factor) function, is dependent on the HIRAN domain [50], which also promotes the HLTF-dependent fork reversal, playing roles in DNA damage tolerance [51]. One of the main tasks of the DNA damage tolerance pathway is to minimize fork stalling, pushing for the bypass of replication blocks [52]. In plants, the HIRAN domain was investigated in relation to AtRAD5A, a DNA translocase that catalyzes the fork regression, and was shown to be able to bind to branched DNA structures and promote DNA repair [53]. However, all the cited literature refers to the HIRAN-SMARCA type of architecture, while no information on the HIRAN-HKD is available in plants. Based on the assumption that the HIRAN domain could act as a sensor to initiate the repair processes at damaged DNA checkpoints [49], we hypothesize that the presence of this motif in the Tdp1β protein might sustain the early response of the gene to abiotic stress conditions. Nonetheless, further experimental studies, such as targeted modifications of the *Tdp1β* HIRAN domain, are needed to prove this theory.

## Supplementary Materials

The following are available online at www.mdpi.com/2073-4425/8/11/305/s1, Table S1: *Arabidopsis thaliana AtTdp1α, AtTdp1β*, and *AtTOP1α, AtTOP1β* gene expression profiles retrieved from the AtGenExpress microarray abiotic stress dataset. Data are presented as the mean of three replicates ± standard deviation, Table S2: *Medicago truncatula MtTdp1α, MtTdp1β*, and *MtTOP1α, MtTOP1β* gene expression profiles obtained from qRT-PCR analysis. Data are presented as the mean of three replicates ± standard deviation, Table S3: Promoter *cis*-elements identified using the PlantCARE online tool. The function of each *cis*-element is specified, alongside with the frequency of presence in the promoter regions of *AtTdp1α*, *AtTdp1β, MtTdp1α*, and *MtTdp1β*.
